# Identification of immune protective genes of *Eimeria maxima* through cDNA expression library screening

**DOI:** 10.1186/s13071-017-2029-4

**Published:** 2017-02-16

**Authors:** XinChao Yang, MengHui Li, JianHua Liu, YiHong Ji, XiangRui Li, LiXin Xu, RuoFeng Yan, XiaoKai Song

**Affiliations:** College of Veterinary Medicine, Nanjing Agricultural University, Nanjing, Jiangsu 210095 People’s Republic of China

**Keywords:** *Eimeria maxima*, cDNA expression library, Immune protective gene

## Abstract

**Background:**

*Eimeria maxima* is one of the most prevalent *Eimeria* species causing avian coccidiosis, and results in huge economic loss to the global poultry industry. Current control strategies, such as anti-coccidial medication and live vaccines have been limited because of their drawbacks. The third generation anticoccidial vaccines including the recombinant vaccines as well as DNA vaccines have been suggested as a promising alternative strategy. To date, only a few protective antigens of *E. maxima* have been reported. Hence, there is an urgent need to identify novel protective antigens of *E. maxima* for the development of neotype anticoccidial vaccines.

**Methods:**

With the aim of identifying novel protective genes of *E. maxima*, a cDNA expression library of *E. maxima* sporozoites was constructed using Gateway technology. Subsequently, the cDNA expression library was divided into 15 sub-libraries for cDNA expression library immunization (cDELI) using parasite challenged model in chickens. Protective sub-libraries were selected for the next round of screening until individual protective clones were obtained, which were further sequenced and analyzed.

**Results:**

Adopting the Gateway technology, a high-quality entry library was constructed, containing 9.2 × 10^6^ clones with an average inserted fragments length of 1.63 kb. The expression library capacity was 2.32 × 10^7^ colony-forming units (cfu) with an average inserted fragments length of 1.64 Kb. The expression library was screened using parasite challenged model in chickens. The screening yielded 6 immune protective genes including four novel protective genes of EmJS-1, EmRP, EmHP-1 and EmHP-2, and two known protective genes of EmSAG and EmCKRS. EmJS-1 is the selR domain-containing protein of *E. maxima* whose function is unknown*.* EmHP-1 and EmHP-2 are the hypothetical proteins of *E. maxima.* EmRP and EmSAG are rhomboid-like protein and surface antigen glycoproteins of *E. maxima* respectively, and involved in invasion of the parasite.

**Conclusions:**

Our results provide a cDNA expression library for further screening of T cell stimulating or inhibiting antigens of *E. maxima.* Moreover, our results provide six candidate protective antigens for developing new vaccines against *E. maxima.*

**Electronic supplementary material:**

The online version of this article (doi:10.1186/s13071-017-2029-4) contains supplementary material, which is available to authorized users.

## Background

Avian coccidiosis is the disease caused by protozoan parasites of the genus *Eimeria* [1, 2]. *Eimeria maxima* is one of the seven *Eimeria* species that infects domestic chickens and results in severe lesions of the small intestine, low efficiency of feed utilization and weight loss [3, 4]. The global economic losses due to avian coccidiosis are more than 3 billion US dollars per year [2, 5, 6]. Control strategies against avian coccidiosis still rely heavily on anti-coccidial medication and live vaccines [1, 4]. However, the application of coccidiostats has been limited because of the rapid emergence of drug resistance and increasing consumer concerns about drug residues in food [7]. Live vaccines have inherent drawbacks such as their limited production, potential reversion to virulence and high cost of manufacture [2, 6, 8]. The search for new approaches for coccidiosis control turned towards third generation anticoccidial vaccines including recombinant vaccines as well as DNA vaccines [4, 6, 9–13]. To date, only a few protective antigens of *E. maxima* have been reported. The lack of candidate protective antigens represents a considerable bottleneck in developing new vaccines against this parasite [14–16]. Hence, finding novel protective antigens of *E. maxima* is urgently required for the future development of univalent vaccines and a multivalent vaccine to protect against infections with multiple *Eimeria* species. With the aim of identifying novel protective genes of *E. maxima*, in the present study, a cDNA expression library of *E. maxima* sporozoites was constructed using Gateway technology. Subsequently, the protective genes of *E. maxima* were screened using cDELI in parasite challenge model and the biological characters of protective genes were analyzed.

## Methods

### Vector, parasite and animals

Eukaryotic expression vector pVAX1 was purchased from Invitrogen (Carlsbad, California, USA). Sporulated oocysts of *E. maxima* (Jiangsu) were collected 7 days prior to the challenge infection. The purity of *E. maxima* was determined with ITS1-PCR as described previously [16, 17]. New-hatched Hy-Line layer chickens (commercial breed W-36) were raised in a sterilized room under coccidia-free conditions until the end of the experiment. Food and water without anti-coccidial drugs were available.

### RNA isolation for library construction

Sporozoites of *E. maxima* were purified from sporulated oocysts on DE-52 anion exchange columns using a previously described protocol [18, 19]. Total RNA was extracted from *E. maxima* sporozoites using E.Z.N.A.® Total RNA Kit Maxi Kit (OMEGA, Norcross, Georgia, USA). Subsequently, mRNA was purified with FastTrack® MAG mRNA Isolation Kits (Invitrogen). The quality of the isolated total RNA and mRNA were determined by denaturing agarose gel electrophoresis.

### Construction of cDNA entry library of *E. maxima*

The cDNA entry library was constructed using CloneMiner™ II cDNA Library Construction Kit (Invitrogen) following manufacturer’s protocol (see Additional file 1: Protocol for constructing entry library). To evaluate the titer of cDNA entry library, 50 μl of the 1000-fold diluted library bacteria were cultured overnight at 37 °C on LB plates containing100 μg/ml ampicillin. After that, colonies on the plate were counted. The titer was calculated as follows: colony-forming units (cfu)/ml = colonies on plate × dilution factor/volume plated (ml). Total cfu of the library = titer (cfu/ml) × total volume of cDNA library (ml). To determine the insert size of the library, 24 random clones were amplified by PCR with universal primers (5′-TCC CAG TCA CGA CGT TGT AAA ACG ACG GCC AGT CTT-3′/5′-AGA GCT GCC AGG AAA CAG CTA TGA CCA TGT AAT ACG ACT C-3′) targeting the pDONR222 vector. The PCR program are as following: 95 °C for 5 min with an initial denaturation, 35 cycles of 94 °C for 30 s, 58 °C for 30 s and 72 °C for 2 min, after 35 cycles, 72 °C for 5 min.

### Construction of cDNA expression library for *E. maxima*

Prior to the construction of a cDNA expression library for *E. maxima*, we ligated the recombinant gene attR1-ccdB-attR2 into the expression vector pVAX1 to construct a Gateway system compatible vector pVAX1-DEST. Briefly, the *attR1*-*ccdB*-*attR2* gene was amplified from the pDEST32 vector (Invitrogen) by PCR using the HindIII/XhoI-flanked primers (F/R 5′-GAC GAC AAG CTT CTG TAT CGT CGA GGT CGA ATC AAA CAA G-3′/5′-TCA TCC TCG AGT ACT TAC TTA GCG GCC ATC AAA CCA C-3′, restriction enzyme of HindIII/XhoI are underlined). The PCR product was digested with restriction enzymes HindIII/XhoI and ligated into the pVAX1 vector to produce Gateway system compatible vector pVAX1-DEST.

Afterwards, the LR recombination reaction was performed to transfer the cDNA entry library into the expression vector pVAX1-DEST using CloneMiner™ II cDNA Library Construction Kit (Invitrogen). The reaction products were transformed into ElectroMAX™ DH10B™ T1 competent cells, producing the cDNA expression library. The cDNA library titer was determined using plating assay described in entry library construction. The similar PCR detection of recombinant fragment size was performed with primers (CMV-F, 5′-CGC AAA TGG GCG GTA GGC GTG-3′ and BGH, 5′-TGG CAA CTA GAA GGC ACA GTC GAG G-3′) of pVAX1-DEST vector.

### Test of the cDNA expression library by PCR amplification of known *E. maxima* genes

To test the representativeness of the cDNA expression library, 7 available *E. maxima* genes in our lab were amplified from the constructed cDNA expression library of *E. maxima.* Briefly, 10 μl of the cDNA expression library was inoculated into 5 ml of broth culture medium which was grown to an OD600 of 1, before the plasmid DNA was isolated from the culture. With the isolated DNA as template, the *E. maxima* genes of MIC3-1, MIC3-2, MIC3-3, MIC2, MIC7, MIC5 and AMA1 were amplified by PCR using specific primers (see Additional file 2: Table S1). The PCR products were analysed by 1% agarose gel electrophoresis.

### The first round of cDNA expression library screening

#### Preparation of plasmid DNA for library screening

Ten microlitres of the cDNA expression library was divided into 15 first-level sub-libraries and plasmid DNA was isolated from each of the sub-libraries for screening. Briefly, 10 μl of cDNA expression library was diluted into 3 ml of LB broth and plated onto 15 LB agar plates (each plate representing an individual sub-library) containing 50 μg/ml kanamycin (200 μl of each plate). Next, the plates were incubated at 37 °C for 16–18 h. All resulting clones were then transferred into 150 ml broth culture medium and incubated at 30 °C for 16–18 h. Plasmid DNA was then isolated from each sub-library using a High Pure Maxi Plasmid Kit (TIANGEN, Beijing, China) following manufacturer’s instructions. Meanwhile, the plasmid DNA of empty vector pVAX1 was prepared. The concentration of the plasmid DNA was measured by NanoDrop 2000 spectrophotometer and stored at -20 °C for further use.

#### Immunization and challenge infection

At 14 days of age, 360 chickens were weighed individually and randomly distributed into 15 experimental groups and 3 control groups of 20 chickens in each. As shown in Table 1, the experimental groups were the 15 sub-library immunized groups, the 3 control groups were pVAX1 vector control, unchallenged control and challenged control group. Experimental groups were immunized with library plasmid DNA by intramuscular injection in legs at a dose of 100 μg. The vector control group was immunized with 100 μg of vector pVAX1, whereas the challenged and unchallenged controls were injected with PBS. A booster immunization was given by the same method as the primary immunization 7 days later. After 1 week of booster immunization, chickens were weighed individually and challenged orally with 1.5 × 10^5^ *E. maxima* oocysts except the unchallenged control group. The chickens were weighed individually and sacrificed by cervical dislocation 7 days post-challenge. Average body-weight gain, survival rate, decreased oocyst output, lesion score, and anticoccidial index (ACI) of each group were calculated as described in the evaluation of protection.

**Table 1:** Protective efficacy of the 15 sub-libraries in the first round of cDNA expression library screening

Group	Average body-weight gain (g)	Relative body-weight gain (%)	Mean lesion scores	Decreased oocyst output (%)	ACI
Unchallenged control	86.59 ± 18.89^f^	100	0 ± 0^a^	100	200
Challenged control	23.50 ± 13.29^a^	25.14	3.10 ± 0.27^f^	0	54.14
pVAX1 control	26.26 ± 10.90^ab^	27.88	2.50 ± 0.31^e^	6.28	62.88
Pool 1	41.67 ± 15.75^bcd^	47.16	1.43 ± 0.40^ab^	49.54	127.79
Pool 2	69.50 ± 25.85^ef^	79.37	1.45 ± 0.45^ab^	60.27	154.79
Pool 3	80.57 ± 12.85^f^	90.80	1.22 ± 0.46^a^	70.57	168.57
Pool 4	58.81 ± 18.80^de^	66.06	2.40 ± 0.47^de^	57.16	137.06
Pool 5	56.90 ± 15.33^cde^	64.60	2.00 ± 0.57^cde^	53.00	139.6
Pool 6	59.38 ± 19.75^de^	65.99	1.94 ± 0.68^cd^	66.55	141.55
Pool 7	82.49 ± 25.46^f^	89.23	1.30 ± 0.59^ab^	68.67	171.16
Pool 8	77.67 ± 20.64^f^	85.52	1.22 ± 0.48^a^	74.59	168.27
Pool 9	49.66 ± 9.97^cd^	52.67	1.42 ± 0.53^ab^	62.03	133.39
Pool 10	57.73 ± 20.12^de^	65.38	2.40 ± 0.45^de^	54.27	136.38
Pool 11	40.60 ± 21.94^abc^	46.85	1.75 ± 0.51^bc^	46.37	124.35
Pool 12	58.89 ± 21.98^de^	64.64	2.10 ± 0.39^cde^	51.96	138.64
Pool 13	24.93 ± 11.20^ab^	26.88	2.48 ± 0.60^e^	15.53	97.07
Pool 14	58.70 ± 22.94^de^	63.45	2.12 ± 0.52^cde^	50.812	137.2
Pool 15	31.68 ± 9.17^ab^	36.41	1.42 ± 0.67^ab^	40.37	117.18

*Note*: Significant difference (*P* < 0.05) between numbers with different letters; non-significant difference (*P* > 0.05) between numbers with the same letter

#### Evaluation of protection

Several criteria were employed for evaluating the efficacy of DNA immunization with the expression library including survival rate, lesion score, body-weight gain, decreased oocyst output and ACI. The equations for calculating the criteria were shown in Additional file 3: Equations of criteria for evaluating the efficacy of DNA immunization with the expression library. Any sub-library with the ACI ≥ 160 was considered protective [20, 21]. Body-weight gain and lesion scores were expressed as the mean ± standard deviation (SD) and statistical analysis was performed using the SPSS statistical package (IBM SPSS Statistics 19). Differences between groups were tested with the one-way ANOVA Duncan test and were considered significant at *P* < 0.05.

### The further rounds screening of the individual protective clone

The second round screening was performed according to the result of the first round screening. Briefly, the protective first-level sub-library was divided into several second-level sub-libraries. The immunization and challenge experiment was carried out to determine the protective second-level sub-library. The experimental design and efficacy evaluation was same as the first round screening. A third, fourth and even fifth round of screening was performed until the individual protective clone was obtained, following the experimental design and efficacy evaluation described in the first round screening.

### DNA sequencing and sequence analysis

The protective clones were sequenced by Invitrogen Company (Shanghai, China). The open reading frame (ORF) of the protective antigen genes was determined with DNASTAR software and ORF Finder (https://www.ncbi.nlm.nih.gov/orffinder/). The ORF and predicted protein sequence of the antigen gene was blasted with NCBI (the National Center for Biotechnology Information, https://blast.ncbi.nlm.nih.gov/Blast.cgi) and ToxoDB database (www.toxodb.org). The T cell epitope motif and antigen index were predicted using DNASTAR software.

## Results

### Construction of *E. maxima* cDNA expression library

**Fig. 1:**
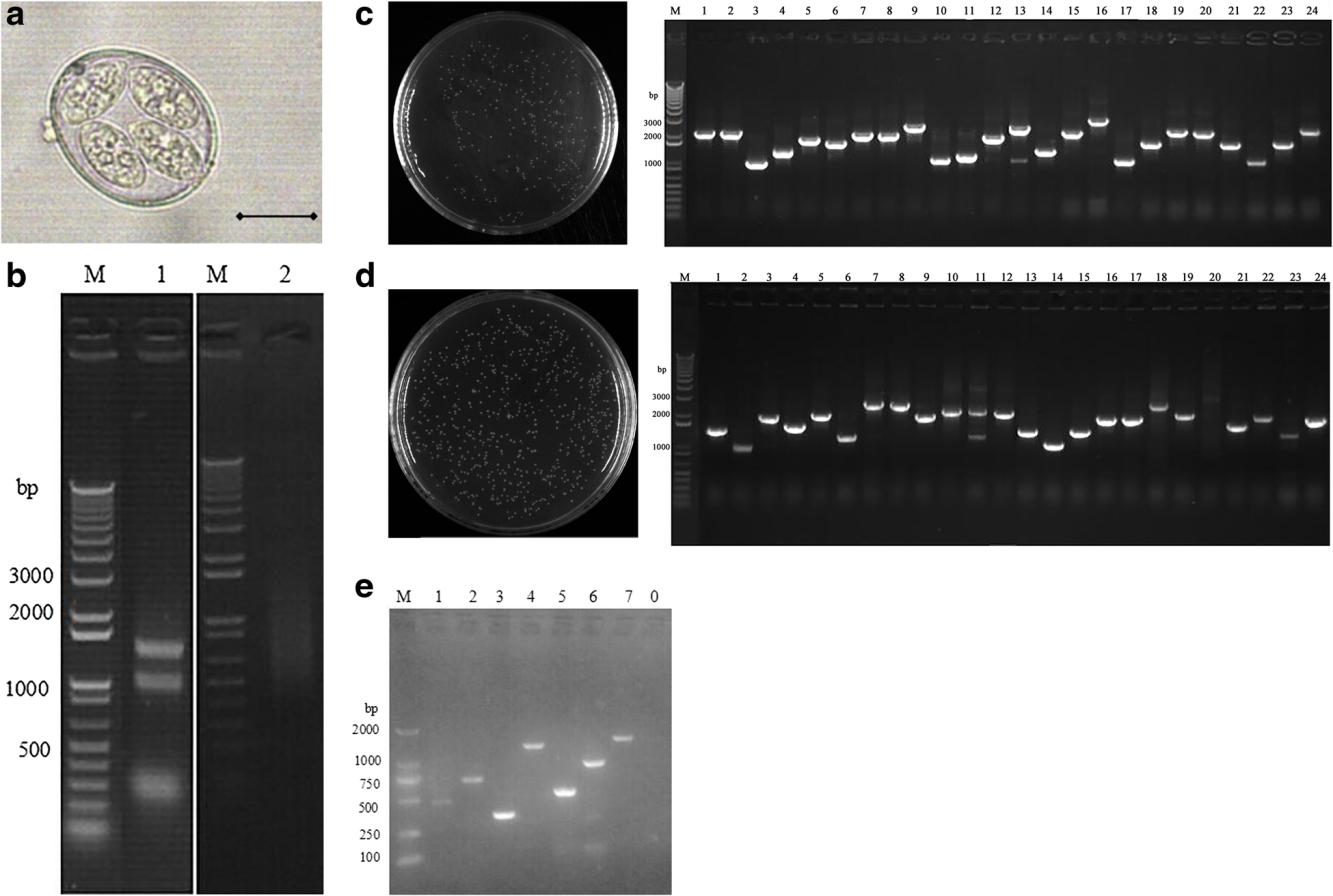
Construction procedure of * E. maxima* cDNA expression library. **a** Sporulated oocyst of *E. maxima. Scale-bar*: 10 μm. **b** Quality determination of the isolated total RNA and mRNA of *E. maxima* by denaturing agarose gel electrophoresis. **c** Titer and insert size evaluation of the entry library using plating assay and PCR assay. **d** Titer and insert size evaluation of the cDNA expression library using plating assay and PCR assay. **e** Amplification of 7 known genes from the cDNA expression library

Our study adopted Gateway technology for library construction with the modified pVAX1 vector. Figure 1a shows sporulated oocysts of *E. maxima*. The integrity and purity of total RNA extracted from the sporozoites were detected by 1% denaturing agarose gel electrophoresis (Fig. 1b, Lane 1) and nucleic acid analyzer (Thermo nanodrop) and purified mRNA were performed for the same analysis (Fig. 1b, Lane 2). As shown in Fig. 1b (Lane 1), electrophoresis clearly revealed 3 bands of 28S, 18S, and 5S of total RNA. Furthermore, the brightness of 28 s was about 2 times of 18 s, which indicated good integrity of the total RNA (Fig. 1b, Lane 2). The quantity of the total RNA was about 600 μg and the A260/A280 value at 1.92, which indicated good purity of the total RNA. The purified mRNA appeared excellent quality with an A260/A280 value of 2.32, whereas the total mRNA quantity was approximately 8.83 μg.

The cDNA entry library titer was determined by serial dilution using plating assay. After growing overnight, 230 clones were counted on the plate (Fig. 1c). According to the equations, the titer of the plate was calculated as 4.6 × 10^6^ cfu, and the total clones of the entry were 9.2 × 10^6^. Insert size of the library was detected by PCR, and the positive bands were generated from all the randomly picked 24 clones. Furthermore, the insert size ranged from 0.9 to 2.8 Kb with an average size of 1.63 Kb (Fig. 1c). These data indicated that the entry library was well represented and could be applied further for the construction of expression library. The cDNA expression library was evaluated in the same way. The results showed that the expression library capacity was 2.32 × 10^7^ cfu. The length of insert was ranged from 1 to 3 Kb with an average size of 1.64 Kb (Fig. 1d).

### Test of the cDNA expression library by PCR amplification of known *E. maxima* genes

To test the representativeness of the cDNA expression library, 7 *E. maxima* genes with different sizes were amplified from the expression library*.* As showed in Fig. 1e, PCR revealed 7 bands of 450, 684, 336, 1275, 519, 888 and 1422 bp. The bands were consistent with the sizes of *E. maxima* genes MIC3-1, MIC3-2, MIC3-3, MIC2, MIC7, MIC5 and AMA1, respectively.

### Screening of cDNA expression library

#### The first round screening

The cDNA expression library was partitioned into 15 sub-libraries (termed pool 1–15) with 200–300 clones per pool. Two-week-old chickens were immunized with the isolated plasmid DNA of the 15 sub-libraries to compare their protective efficacies. As shown in Table 1, except pool 13, immunization with the other 14 pools resulted in alleviated enteric lesion, higher weight gain and decreased oocyst output as compared to the pVAX1 vector control group and challenged control group. Pools 3, 7 and 8 induced the highest decreased oocyst output of 70.57, 68.67 and 74.59%, which resulted in ACIs of 168.57, 171.16 and 168.27, respectively, indicating effective protection. The combined results indicated that pool 3, 7 and 8 were the protective pools and could be selected for the second round screening of protective clones.

#### The second round screening

Since pool 3, 7 and 8 were the most protective, emphasis was given to identifying the protective component(s) in these pools. As shown in (Table 2), pool 3, 7 and 8 were partitioned into 4 second-level sub-libraries respectively (designated pool 3–1 to 3–4, pool 7–1 to 7–4, pool 8–1 to 8–4) with 50–75 clones per pool. Animal experiments were performed to compare the protective efficacy of the second-level sub-libraries following the experimental design described as the first round screening. The results were shown in Table 2. Pool 8–2 induced the highest decreased oocyst output and ACI of 172.27, indicating that it could be selected for the third round screening of protective clones.

**Table 2:** Protective efficacy of the 12 sub-libraries in the second round of cDNA expression library screening

Group	Average body-weight gain (g)	Relative body-weight gain (%)	Mean lesion scores	Decreased oocyst output (%)	ACI
Unchallenged control	79.97 ± 18.85^f^	100	0 ± 0^a^	100	200
Challenged control	54.34 ± 20.57^bc^	68.40	2.61 ± 0.37^h^	0	102.23
pVAX1 control	52.80 ± 19.10^ab^	71.04	1.65 ± 0.64^bcd^	12.50	114.46
Pool 3–1	42.06 ± 23.21^a^	52.88	2.25 ± 0.55^fg^	51.39	120.38
Pool 3–2	48.79 ± 22.91^a^	63.82	2.50 ± 0.76^gh^	31.11	128.82
Pool 3–3	47.96 ± 22.10^ab^	61.75	1.85 ± 0.36^cde^	51.39	133.25
Pool 3–4	42.81 ± 36.90^ab^	54.88	2.20 ± 0.57^f^	40.56	127.88
Pool 7–1	60.47 ± 28.12^cd^	79.86	1.50 ± 0.48^b^	36.11	144.86
Pool 7–2	54.49 ± 30.97^bc^	69.62	1.57 ± 0.70^bc^	69.44	143.85
Pool 7–3	60.14 ± 16.57^cd^	79.75	1.93 ± 0.45^cde^	70.83	150.42
Pool 7–4	60.58 ± 21.90^cd^	82.32	1.64 ± 0.49^bcd^	52.78	155.85
Pool 8–1	64.21 ± 15.77^de^	82.55	1.63 ± 0.61^bcd^	50.00	156.22
Pool 8–2	76.23 ± 18.14^ef^	94.77	1.75 ± 0.54^bcde^	88.89	172.27
Pool 8–3	60.58 ± 19.20^cd^	81.82	2.00 ± 0.44^def^	31.94	141.82
Pool 8–4	69.19 ± 21.47^def^	83.58	2.05 ± 0.34^e^	56.94	152.99

*Note*: Significant difference (*P* < 0.05) between numbers with different letters; non-significant difference (*P* > 0.05) between numbers with the same letter

#### The third round screening

The protective pool 8–2 (63 clones) was partitioned into 9 third-level sub-libraries (designated pool 8–2–1 to 8–2–9) with 7 clones per pool. The results of protective efficacies of each clone were shown in (Table 3). Pool 8–2–2 and 8–2–8 induced the ACIs of 164.54 and 163.43, respectively, indicating that pool 8–2–2 and 8–2–8 could be selected for the fourth round screening of protective individual clones.

**Table 3:** Protective efficacy of the 9 sub-libraries in the thrid round of cDNA expression library screening

Group	Average body-weight gain (g)	Relative body-weight gain (%)	Mean lesion scores	Decreased oocyst output (%)	ACI
Unchallenged control	98.26 ± 13.09^e^	100	0 ± 0^a^	100.00	200
Challenged control	37.72 ± 41.47^a^	40.91	3.05 ± 0.84^f^	0.00	70.41
pVAX1 control	41.47 ± 27.85^ab^	43.81	2.60 ± 1.16^e^	17.95	77.81
Pool 8–2–1	41.98 ± 26.55^ab^	45.34	2.00 ± 1.19^d^	75.64	120.34
Pool 8–2–2	79.85 ± 40.00^d^	84.74	1.02 ± 1.25^b^	61.54	164.54
Pool 8–2–3	54.62 ± 42.72^bc^	59.28	2.12 ± 1.17^d^	65.38	128.08
Pool 8–2–4	44.65 ± 30.68^ab^	46.35	2.30 ± 1.12^de^	71.79	113.35
Pool 8–2–5	43.87 ± 28.02^ab^	47.15	1.37 ± 1.07^bc^	43.59	113.45
Pool 8–2–6	51.47 ± 25.97^bc^	55.58	2.10 ± 1.29^d^	62.82	124.58
Pool 8–2–7	55.07 ± 26.63^c^	56.80	2.32 ± 1.09^de^	64.10	123.6
Pool 8–2–8	73.93 ± 30.41^d^	82.93	1.45 ± 1.35^c^	84.62	163.43
Pool 8–2–9	45.56 ± 38.25^abc^	50.20	2.55 ± 0.95^e^	62.82	114.7

*Note*: Significant difference (*P* < 0.05) between numbers with different letters; non-significant difference (*P* > 0.05) between numbers with the same letter

#### The fourth round screening

All the individual clones from the two positive pools (pool 8–2–2 and 8–2–8) were designated as clone 8–2–2–1 to 8–2–2–7 and 8–2–8–1 to 8–2–8–7 respectively (Table 4). The results of protective efficacies of the 14 single clones were shown in Table 4. Six individual clones induced ACIs > 160 namely, 8–2–2–2 (162.97), 8–2–2–5 (167.21), 8–2–8–1 (162.22), 8–2–8–2 (160.02), 8–2–8–3 (160.02) and 8–2–8–6 (160.74).

**Table 4:** Protective efficacy of the 14 single clones in the fourth round of cDNA expression library screening

Group	Average body-weight gain (g)	Relative body-weight gain (%)	Mean lesion scores	Decreased oocyst output (%)	ACI
Unchallenged control	57.61 ± 9.47^f^	100	0 ± 0^a^	100.00	200
Challenged control	36.89 ± 11.89^ab^	63.68	2.63 ± 0.63^fg^	0.00	97.30
pVAX1 control	37.42 ± 6.82^abc^	60.29	1.80 ± 0.38^cdef^	8.91	102.24
Clone 8–2–2–1	41.76 ± 18.49^abcde^	69.09	2.81 ± 1.50^g^	56.44	130.97
Clone 8–2–2–2	48.83 ± 12.79^cdef^	81.12	0.81 ± 0.62^b^	51.49	162.97
Clone 8–2–2–3	40.12 ± 13.95^abcd^	64.47	2.36 ± 1.24^defg^	13.86	100.79
Clone 8–2–2–4	43.26 ± 14.30^abcde^	73.50	2.84 ± 1.21^g^	14.85	105.08
Clone 8–2–2–5	56.63 ± 26.03^f^	93.89	1.66 ± 0.93^bcde^	56.44	167.21
Clone 8–2–2–6	38.82 ± 11.68^abcd^	63.52	1.92 ± 1.32^cdef^	23.76	104.31
Clone 8–2–2–7	39.88 ± 18.61^abcd^	66.43	2.78 ± 1.42^g^	18.81	98.54
Clone 8–2–8–1	50.04 ± 19.32^def^	84.86	1.26 ± 0.92^bc^	52.48	162.22
Clone 8–2–8–2	48.27 ± 16.57^bcdef^	80.03	1.50 ± 1.31^bcd^	82.18	160.02
Clone 8–2–8–3	49.52 ± 18.54^def^	85.80	1.57 ± 1.75^bcd^	72.28	160.02
Clone 8–2–8–4	34.84 ± 14.24^a^	60.28	1.52 ± 1.24^bcd^	15.84	105.02
Clone 8–2–8–5	46.87 ± 12.41^bcdef^	82.19	1.47 ± 1.19^bc^	66.34	157.46
Clone 8–2–8–6	52.41 ± 15.10^ef^	89.07	1.83 ± 1.24^cdef^	60.40	160.74
Clone 8–2–8–7	39.89 ± 11.89^abcd^	68.08	2.46 ± 1.14^efg^	35.64	123.40

*Note*: Significant difference (*P* < 0.05) between numbers with different letters; non-significant difference (*P* > 0.05) between numbers with the same letter

### DNA sequence analysis

After four rounds screening, 6 individual protective clones were identified. The positive clone 8–2–2–2 shared 76% identity in amino acids with hypothetical protein of *E. maxima* (GenBank: CDJ56976.1) and was named as EmHP-1. The positive clone 8–2–2–5 shared 100% identity in amino acids with SAG family member of *E. maxima* (GenBank: CDJ60815.1) and was named as EmSAG. The positive clone 8–2–8–1 shared 91 and 84% identity in amino acids with *Eimeria tenella* rhomboid-like protein (GenBank: ABC50099.1) and *E. maxima* rhomboid family domain-containing protein, putative (GenBank: CDJ59262.1) respectively, and was named as EmRP. The positive clone 8–2–8–2 shared 70% identity in amino acids with *E. maxima* hypothetical protein (GenBank: CDJ61108.1) and was named as EmHP-2. The positive clone 8–2–8–3 shared 100% identity in amino acids with *Eimeria maxima* CAMP-dependent protein kinase regulatory subunit (GenBank: CDJ61187.1) and was named as EmCKRS. The positive clone 8–2–8–6, named as EmJS-1, shared 79% identity in amino acids with hypothetical protein of *Eimeria_necatrix_*Houghton (ToxoDB: ENH_00014740) and no identity with the known gene of *E. maxima*.

Characterizations of these 6 protective clones were shown in (Table 5). Complete ORFs were included within the 6 antigen genes separately (Table 5, Additional file 4: Figure S1). Prediction of T cell epitope motif and antigen index revealed that the identified antigens are abundant in T cell epitope motifs and regions with high antigenic index (Table 5, Additional file 5: Figure S2). EmHP-1, EmRP, EmHP-2 and EmJS-1 are novel genes of *E. maxima,* their nucleotide sequences and amino acids have been submitted to GenBank with the accession numbers of KR868754.1, KR815509, KR868755.1 and KR868753.1, respectively (Table 5).

**Table 5:** Characterizations of the six protective clones screened from the cDNA expression library of *E. maxima*

Clones	Designated name	Insert size (bp)	ORF (bp)	Predicted molecular mass (kDa)	Isoelectric point	Predicted T cell epitope motifs	Regions with high antigenic index	GenBank accession number
8–2–2–2	EmHP–1	1162	957	35.49	5.05	4–13, 84–88, 96–99, 129–132, 151–154, 175–178, 197–201, 240–243, 250–260, 271–278, 281–284	10–33, 38–48, 69–145, 149–167, 173–217, 229–243	KR868754.1
8–2–2–5	EmSAG	1076	708	24.73	4.81	22–25, 39–42, 45–48, 63–66, 79–82, 106–109, 122–126, 135–138, 160–163, 172–179, 188–192, 200–203, 214–217	34–42, 45–50, 53–66, 68–88, 94–101, 115–124, 127–136, 144–151, 156–175	CDJ60815.1
8–2–8–1	EmRP	1004	774	28.34	8.27	8–11, 19–22, 60–64, 66–70, 90–97, 150–153, 195–198, 222–228, 245–249	1–10, 14–23, 27–35, 54–62, 105–118, 165–175, 212–220, 226–234	KR815509
8–2–8–2	EmHP–2	1261	753	27.91	5.01	29–32, 114–118, 133–139, 149–155, 160–163, 174–177, 192–195, 198–201, 225–232	11–27, 14–23, 32–43, 71–124, 135–152, 166–190, 243–2247, 226–234	KR868755.1
8–2–8–3	EmCKRS	1150	930	32.97	4.33	2–5, 7–10, 23–27, 59–63, 76–79, 139–142, 170–174, 208–211, 219–222, 229–233, 241–244, 247–250, 268–271, 275–279, 285–289	1–17, 24–42, 55–80, 94–109, 118–221, 232–258, 270–284, 292–309	CDJ61187.1
8–2–8–6	EmJS–1	1233	603	22.26	8.33	73–76, 78–81, 125–128, 132–136, 157–160, 163–166, 179–182, 192–195	54–64, 67–90, 110–118, 123–132, 137–145, 160–173, 180–200, 292–309	KR868753.1

## Discussion

The expression library immunization (ELI) has proven to be a useful strategy to identifying protective gene pools for novel vaccine candidates, even when little is known about the possible antigenic targets [22, 23]. The cDELI, based on a large number of cDNA clones, has additional advantages over genomic immunization approaches, because a cDNA expression library represents only those genes that are being expressed and the selection of stage-specific antigens is possible. The use of cDELI could be particularly attractive for pathogens with complicated life-cycles and large genomes [24]. To date, cDELI has discovered protective genes or gene pools from a diverse set of bacterial, fungal, and parasitic pathogens [23–27]. In this research, we used cDELI to screen the protective genes of *E. maxima* and successfully obtained six protective genes.

As effective protection is the key characteristic of a practical vaccine, ELI was originally designed with the intention of using actual pathogen challenge as the screening criterion [22, 23]. In this study, we screened the protective genes using actual parasite challenge model in chickens and the six screened genes did provide effective protection against *E. maxima*. The DNA sequence analysis revealed that the six genes are abundant in predicted T cell epitope motifs and regions with high antigenic index. In our subsequent study, we immunized chickens with the identified genes and evaluated the level of cytokines, T cell subtype and IgG of the immunized chickens. The results revealed that the immunization with the genes induced significantly enhanced T cell response and antibodies in the immunized chickens (unpublished data), compared with the control chickens, which are consistent with the DNA sequence analysis and the effective protection of these genes.

In the current study, only six protective genes were obtained through cDELI. The number of protective genes isolated through cDELI was also limited in previous studies. For example, Ivey et al. [25] isolated only one protective gene of *Coccidioides immitis* by cDELI. Tekiel et al. [27] obtained 28 protective genes of *Trypanosoma cruzi* from a trypomastigote cDNA expression library. Huntley et al. [28] identified 26 protective genes of *Mycobacterium avium paratuberculosis.* There are several reasons why the number of protective genes isolated using cDELI is often small. The following explanations might answer this question. First, since the genome of *Eimeria* spp*.* is estimated to be between 55 and 60 Mbp in size, encoding 8000–9000 genes (http://www.genedb.org/Homepage/Etenella), it is very difficult to include all genes in the cDNA library, and some protective genes might be lost during the library construction, such as two protective antigens AMA-1 and IMP-1 described by Blake et al. [14]. Secondly, another explanation may be the weakness of ELI approach. The simultaneous expression of many antigens could lead to antigenic competition. For example, some antigens are known to be the focus of immune responses, while others can induce immunological non-responsiveness which could mask the effective antigens in cDELI [29]. The effective antigens also could be masked by the dilution effect during cDELI. For example, if one sub-library contains 100 plasmids during cDELI, each individual plasmid will be delivered in 1/100th of the maximal dose of DNA and will therefore generate a weaker response than if delivered by itself at the highest dose [23]. This could also be due to the fact that the gene length probably alters the cloning/transformation efficiency. Some longer transcripts may clone less efficiently than smaller transcripts. Thirdly, we used ACI as a screening criterion, a synthetic criterion including weight gain, survival rate, oocysts output and lesion score. Since ACIs of some clones/sub-libraries were very close to 160, one possible way to increase the number of protective antigens is to pick clones with a lower ACI and then test them for the immunologic parameters.

In this study, we identified four novel protective antigen genes (EmJS-1, EmRP, EmHP-1 and EmHP-2) and two known antigen genes (EmSAG and EmCKRS). EmJS-1 is a selR domain-containing protein of *E. maxima* whose function is unknown*.* Rhomboid-like protein is involved in shedding adhesions from the surface of several apicomplexan parasites during motility and host cell entry by cleaving their substrates microneme proteins within their transmembrane domains [30, 31]. However, rhomboid protein functions with different substrate specificities [32]. In *Toxoplasma gondii*, TgROM1, TgROM2 and TgROM5 cleaved the transmembrane (TM) domain of *Drosophila* Spitz. TgROM2 cleaved the TM domains of TgMIC2 and TgMIC12 [33]. TgROM4 participated in processing of surface adhesions including TgMIC2, AMA1 and TgMIC3 [34]. In *Eimeria tenella*, EtROM3 was involved in the cleavage of EtMIC4 [31]. The activity and substrate specificity of *E. maxima* rhomboid (EmRP) has not been reported. Surface antigen glycoproteins (SAGs) of *E. tenella* are implicated in host-parasite interactions, where they are thought to be involved in the initial attachment of the parasite to the host [35]. However, the functions of these genes in *E. maxima* remain unknown. Therefore, further researches are needed to evaluate the functions of the six protective antigens identified in this study.

## Conclusions

This study identified six protective genes of *E. maxima* through cDNA expression library construction and screening. Of the six protective genes, four are new and include EmJS-1, EmRP, EmHP-1 and EmHP-2. The remaining protective genes, EmSAG and EmCKRS, are known. EmJS-1 is the selR domain-containing protein of *E. maxima* whose function is unknown*.* EmHP-1 and EmHP-2 are the hypothetical proteins of *E. maxima* and EmRP and EmSAG are implicated in the invasion of the parasite. Our results provide a cDNA expression library for the further screening of T cell stimulating or inhibiting antigens of *E. maxima.* Moreover, our data provided six candidate protective antigens for developing new vaccines against *E. maxima.*

## Additional files

Additional file 1: Protocol for constructing entry library. (DOCX 15 kb)
Additional file 2: Table S1. Specific primers of 7 known *E. maxima* genes used in representativeness test of the cDNA expression library. (DOCX 16 kb)
Additional file 3: Equations of criteria for evaluating the efficacy of DNA immunization with the expression library. (DOCX 17 kb)
Additional file 4: Figure S1. Open reading frame and deduced amino acid sequence of the six protective clones screened from the cDNA expression library of *E. maxima*. (TIF 3156 kb)
Additional file 5: Figure S2. T cell epitope motif and antigen index of the six protective clones predicted using DNASTAR software. (TIF 1051 kb)

## Abbreviations

ACI: Anticoccidial index
AMA1: Apical membrane antigen 1
cDELI: cDNA expression library immunization
cfu: Colony-forming units
ELI: Expression library
EmCKRS: *Eimeria maxima:* CAMP-dependent protein kinase regulatory subunit
EmHP-1: *Eimeria maxima* hypothetical protein 1
EmHP-2: *Eimeria maxima* hypothetical protein 2
EmRP: *Eimeria maxima* rhomboid protein
EmSAG: *Eimeria maxima* surface antigen glycoprotein
ITS1-PCR: Polymerase chain reaction based on the internal transcribed spacer 1
MIC1: Microneme 1
MIC2: Microneme 2
MIC3: Microneme 3
MIC5: Microneme 5
MIC7: Microneme 7
NCBI: The National Center for Biotechnology Information
ORF: Open reading frame
PBS: Phosphate buffer saline
PCR: Polymerase chain reaction
S.D.: Standard deviation
TM: Transmembrane

## References

[1] Innes E, Vermeulen A (2006). Vaccination as a control strategy against the coccidial parasites *Eimeria*, *Toxoplasma* and *Neospora*. Parasitology.

[2] Blake DP, Tomley FM (2014). Securing poultry production from the ever-present *Eimeria* challenge. Trends Parasitol.

[3] Song K, Lillehoj H, Choi K, Yun C, Parcells M, Huynh J (2000). A DNA vaccine encoding a conserved *Eimeria* protein induces protective immunity against live *Eimeria acervulina* challenge. Vaccine.

[4] Witcombe DM, Smith NC (2014). Strategies for anti-coccidial prophylaxis. Parasitology.

[5] Williams R (1999). A compartmentalised model for the estimation of the cost of coccidiosis to the world’s chicken production industry. Int J Parasitol.

[6] Dalloul RA, Lillehoj HS (2006). Poultry coccidiosis: recent advancements in control measures and vaccine development. Expert Rev Vaccines.

[7] Clarke L, Fodey TL, Crooks SR, Moloney M, O’Mahony J, Delahaut P (2014). A review of coccidiostats and the analysis of their residues in meat and other food. Meat Sci.

[8] Du A, Wang S (2005). Efficacy of a DNA vaccine delivered in attenuated *Salmonella typhimurium* against *Eimeria tenella* infection in chickens. Int J Parasitol.

[9] Jenkins MC (2001). Advances and prospects for subunit vaccines against protozoa of veterinary importance. Vet Parasitol.

[10] Chapman H (2014). Milestones in avian coccidiosis research. A review. Poultry Sci.

[11] Huang J, Zhang Z, Li M, Song X, Yan R, Xu L (2015). Immune protection of microneme 7 (EmMIC7) against *Eimeria maxima* challenge in chickens. Avian Pathol.

[12] Huang J, Zhang Z, Li M, Song X, Yan R, Xu L (2015). *Eimeria maxima* microneme protein 2 delivered as DNA vaccine and recombinant protein induces immunity against experimental homogenous challenge. Parasitol Int.

[13] Meunier M, Chemaly M, Dory D (2016). DNA vaccination of poultry: the current status in 2015. Vaccine.

[14] Blake DP, Billington KJ, Copestake SL, Oakes RD, Quail MA, Wan KL (2011). Genetic mapping identifies novel highly protective antigens for an apicomplexan parasite. PLoS Pathog.

[15] Jenkins M, Fetterer R, Miska K, Tuo W, Kwok O, Dubey J (2015). Characterization of the *Eimeria maxima* sporozoite surface protein IMP1. Vet Parasitol.

[16] Jenkins M, Miska K, Klopp S (2006). Application of polymerase chain reaction based on ITS1 rDNA to speciate *Eimeria*. Avian Dis.

[17] Haug A, Thebo P, Mattsson JG (2007). A simplified protocol for molecular identification of *Eimeria* species in field samples. Vet Parasitol.

[18] Schmatz DM, Crane MSJ, Murray PK (1984). Purification of *Eimeria* sporozoites by DE-52 anion exchange chromatography. J Protozool.

[19] Klotz C, Gehre F, Lucius R, Pogonka T (2007). Identification of *Eimeria tenella* genes encoding for secretory proteins and evaluation of candidates by DNA immunisation studies in chickens. Vaccine.

[20] McManus EC, Campbell WC, Cuckler AC (1968). Development of resistance to quinoline coccidiostats under field and laboratory conditions. J Parasitol.

[21] Chapman H (1998). Evaluation of the efficacy of anticoccidial drugs against *Eimeria* species in the fowl. Int J Parasitol.

[22] Barry MA, Lai WC, Johnston SA (1995). Protection against mycoplasma infection using expression-library immunization. Nature.

[23] Barry MA, Howell DP, Andersson HA, Chen JL, Singh RA (2004). Expression library immunization to discover and improve vaccine antigens. Immunol Rev.

[24] Manoutcharian K, Terrazas LI, Gevorkian G, Govezensky T (1998). Protection against murine cysticercosis using cDNA expression library immunization. Immunol Lett.

[25] Ivey FD, Magee DM, Woitaske MD, Johnston SA, Cox RA (2003). Identification of a protective antigen of *Coccidioides immitis* by expression library immunization. Vaccine.

[26] Melby PC, Ogden GB, Flores HA, Zhao W, Geldmacher C, Biediger NM (2000). Identification of vaccine candidates for experimental visceral leishmaniasis by immunization with sequential fractions of a cDNA expression library. Infect Immun.

[27] Tekiel V, Alba-Soto CD, González Cappa SM, Postan M, Sánchez DO (2009). Identification of novel vaccine candidates for Chagas’ disease by immunization with sequential fractions of a trypomastigote cDNA expression library. Vaccine.

[28] Huntley J, Stabel J, Paustian M, Reinhardt T, Bannantine J (2005). Expression library immunization confers protection against *Mycobacterium avium* subsp. *paratuberculosis* infection. Infect Immun.

[29] Ulmer JB, Liu MA (1996). ELI’s coming: expression library immunization and vaccine antigen discovery. Trends Microbiol.

[30] Santos JM, Graindorge A, Soldati-Favre D (2012). New insights into parasite rhomboid proteases. Mol Biochem Parasitol.

[31] Zheng J, Gong P, Jia H, Li M, Zhang G, Zhang X (2014). *Eimeria tenella* rhomboid 3 has a potential role in microneme protein cleavage. Vet Parasitol.

[32] Baker RP, Wijetilaka R, Urban S (2006). Two *Plasmodium* rhomboid proteases preferentially cleave different adhesins implicated in all invasive stages of malaria. PLoS Pathog.

[33] Dowse TJ, Pascall JC, Brown KD, Soldati D (2005). Apicomplexan rhomboids have a potential role in microneme protein cleavage during host cell invasion. Int J Parasitol.

[34] Buguliskis JS, Brossier F, Shuman J, Sibley LD (2010). Rhomboid 4 (ROM4) affects the processing of surface adhesins and facilitates host cell invasion by *Toxoplasma gondii*. PLoS Pathog.

[35] Ramly NZ, Rouzheinikov SN, Sedelnikova SE, Baker PJ, Chow YP, Wan KL (2013). Crystallization and preliminary crystallographic analysis of a surface antigen glycoprotein, SAG19, from *Eimeria tenella*. Acta Crystallogr Sect F: Struct Biol Cryst Commun.

