# Supplementary material for: Identification of immune protective genes of Eimeria maxima through cDNA expression library screening
Source: Parasit Vectors. 2017 Feb 16;10:85. doi: 10.1186/s13071-017-2029-4 (PMC5322808; doi:10.1186/s13071-017-2029-4)
Supplement: Additional file 1: — Protocol for constructing entry library. (DOCX 15 kb) [file 13071_2017_2029_MOESM1_ESM.docx]

Protocol for constructing entry library

The purified mRNA was mixed with the Biotin-attB2-Oligo (dT) Primer, 5×First Strand Buffer, DTT, dNTPs and SuperScript™ III reverse transcriptase to synthesize the first strand of cDNA. Next, the reagents of 5×Second Strand Buffer, dNTPs, *E. coli* DNA Ligase, *E. coli* DNA polymerase I and *E. coli* RNase H were added to the first strand of cDNA to synthesize the second strand of cDNA. Subsequently, the attB1 adapter was ligated to the 5΄ end of the double-stranded cDNA, which was size fractionated by column chromatography to remove the small fragments of DNA. The BP (attB×attP) recombination reaction was performed using the attB-flanked cDNA insert with a pDONR™ 222 vector. Subsequently, the BP reaction mix was transformed into ElectroMAX™ DH10B™ competent cells by electroporation. After that, the electroporated cells were incubated at 37℃ for 1 h to obtain the cDNA entry library.
