# Supplementary material for: Identification of immune protective genes of Eimeria maxima through cDNA expression library screening
Source: Parasit Vectors. 2017 Feb 16;10:85. doi: 10.1186/s13071-017-2029-4 (PMC5322808; doi:10.1186/s13071-017-2029-4)
Supplement: Additional file 2: Table S1. — Specific primers of 7 known E. maxima genes used in representativeness test of the cDNA expression library. (DOCX 16 kb) [file 13071_2017_2029_MOESM2_ESM.docx]

**Table S1** Specific primers of 7 known *E. maxima* genes used in representativeness test of the cDNA expression library

| Gene | Primer | Product size/ bp |
| --- | --- | --- |
| MIC3-1 | Forward: 5' ATGAAGCTGTCGCCTTTCTGTCTCG 3' | 450 |
|  | Reverse: 5' CTAAGATGCACCAGTTGTGTACCGTACAT 3' |  |
| MIC3-2 | Forward: 5' TCGGAAGCAGTTCGTCTGGACAGAAGCT 3' | 684 |
|  | Reverse: 5' TTCCGCGAGATAAGTGTTTGGATGAGGT 3' |  |
| MIC3-3 | Forward: 5' TGCACACAGCAAGGAGAAACAGGGA 3' | 336 |
|  | Reverse: 5' CGGAACACCAGGGCAAGTTATTGCA 3' |  |
| MIC5 | Forward: 5' CAATGGGGTTCTCCTTATACCGAGAT 3' | 1275 |
|  | Reverse: 5' GGAGGCGATATAGAAGTAGCATATCTTTC 3' |  |
| MIC7 | Forward: 5' CCGGAATTCATGAGAAGCTTCGGGGCGAT 3' | 519 |
|  | Reverse: 5' CCGCTCGAGTTACGCCGATTGCTCCACATT 3' |  |
| MIC2 | Forward: 5' CCGGAATTCATGGCTCGCGCCCTTTCAT 3' | 888 |
|  | Reverse: 5' CCCCTCGAGCTAGGAGCTGACCGATGTTGTGG 3' |  |
| AMA1 | Forward: 5' ATGTGTGGATTGCGCGCTGCGTT 3' | 1422 |
|  | Reverse: 5' CTAGTAATCTTGGTCAACTAACAC 3' |  |
