# Supplementary material for: Identification of immune protective genes of Eimeria maxima through cDNA expression library screening
Source: Parasit Vectors. 2017 Feb 16;10:85. doi: 10.1186/s13071-017-2029-4 (PMC5322808; doi:10.1186/s13071-017-2029-4)
Supplement: Additional file 3: — Equations of criteria for evaluating the efficacy of DNA immunization with the expression library. (DOCX 17 kb) [file 13071_2017_2029_MOESM3_ESM.docx]

Equations of criteria for evaluating the efficacy of DNA immunization with the expression library

Survival rate was estimated by the number of surviving chickens divided by the number of initial chickens. Body-weight gain of the chickens in each group was determined by subtracting the body weight at the end of the experiment from the body weight at the time of challenge. The lesion score of the chickens from each group was investigated according to the method of Johnson and Reid [1]. Oocysts counts were expressed by the number of oocysts from 1 g of the enteric content from each chicken using McMaster’s counting technique. Decreased oocyst output was calculated as follows: the number of oocysts from positive control chickens-vaccinated chickens/positive control chickens×100%. ACI is a synthetic criterion for assessing the protective effect of a medicine or vaccine and was calculated as follows: (the percentage relative rate of weight gain + the percentage survival rate) - (the lesion index + the oocysts index). ACI is considered "good" at 180 or more, "moderate" between 160-179, and "poor" when below 160 [2, 3].

References:

1. Johnson J, Reid WM. Anticoccidial drugs: lesion scoring techniques in battery and floor-pen experiments with chickens. Exp Parasitol. 1970; 28(1): 30-6.
2. McManus EC, Campbell WC, Cuckler AC. Development of resistance to quinoline coccidiostats under field and laboratory conditions. J Parasitol. 1968; 54(6):1190-3.
3. Chapman H. Evaluation of the efficacy of anticoccidial drugs against *Eimeria* species in the fowl. Int J Parasitol. 1998; 28(7):1141-4.
